# A Reversible Bis(Salamo)-Based Fluorescence Sensor for Selective Detection of Cd^2+^ in Water-Containing Systems and Food Samples

**DOI:** 10.3390/ma11040523

**Published:** 2018-03-29

**Authors:** Jing Hao, Xiao-Yan Li, Yang Zhang, Wen-Kui Dong

**Affiliations:** School of Chemical and Biological Engineering, Lanzhou Jiaotong University, Lanzhou 730070, China; haojingmm@126.com (J.H.); L1401569787@163.com (X.-Y.L.); zhangy8124@163.com (Y.Z.)

**Keywords:** bis(salamo)-type tetraoxime, optical chemosensor, Cd^2+^, detection, test strips

## Abstract

A novel, simple, highly selective, and sensitive fluorescence chemosensor for detecting Cd^2+^ that was constructed from a bis(salamo)-type compound (H_4_L) with two N_2_O_2_ chelating moieties as ionophore was successfully developed. Sensor H_4_L could show fluorescence turn-on response rapidly and significant selectivity to Cd^2+^ over many other metallic ions (Cu^2+^, Ba^2+^, Ca^2+^, K^+^, Cr^3+^, Mn^2+^, Sr^2+^, Co^2+^, Na^+^, Li^+^, Ni^2+^, Ag^+^, and Zn^2+^), and a clear change in color from colorless to yellow that can be very easily observed via the naked eyes in the existence of Cd^2+^, while other metallic ions do not induce such a change. Interestingly, its fluorescent intensity was increased sharply with the increased concentration of Cd^2+^. The detection limit of sensor H_4_L towards Cd^2+^ was down to 8.61 × 10^−7^ M.

## 1. Introduction

Metallic ions play a key role in daily life [1,2,3,4]. Cadmium, which is an essential resource in the earth, is widely used in all kinds of agricultural processes and industry containing chemical industry, electronics industry, nuclear industry semiconducting, quantum dots, phosphate fertilizers, and other fields [5]. However, it should be vigilant that Cd^2+^ is a heavy metallic ion with highly toxic [6]. Some new studies reveal that a considerable amount of cadmium go into the environment from waste water, waste residue, and exhaust gas not only damages the environment, but also endangers human health [7]. Hence, with ever-increasing concern for environment and human health, there is now a much greater demand for the development of a rapid and convenient detection method for Cd^2+^ ion [8,9].

Up until now, with the development of optical sensors for recognizing heavy and transition metal ions in living organisms [10,11], intense efforts have been devoted to the design and synthesis of high sensitivity fluorescence sensors due to the lower cost and rapid response, as well as the easy operability of the fluorescent technique [12,13,14,15,16,17]. According to the relevant literatures, the metallic coordination compounds with salen-type N_2_O_2_ ligands and their corresponding analogues could be used to catalysis [18], nonlinear optical materials and magnetic materials [19,20,21,22,23,24,25,26,27,28], supramolecular architecture [29,30], ions recognition [31,32,33,34,35,36,37,38,39,40], and biological fields, and so forth [41,42,43,44,45,46,47]. Today, researches on the participation of bis(salamo)-type compounds in ion recognition are not to be explored [48,49,50,51,52,53,54,55,56,57,58]. Notably, when compared with most of known fluorescence probes for Zn^2+^ and Cu^2+^, there are relatively few reports on fluorescent probes for Cd^2+^. 

As part of our ongoing interest in the development of new optical (both colorimetric and fluorescence) chemosensor, in the present paper, we report a bis(salamo)-type tetraoxime sensor H_4_L for detecting Cd^2+^ by turn-on response in Tris-phosphate buffer (c = 0.2 M, DMF/H_2_O = 9:1, *v*/*v*, pH = 7.0) solution. The sensor H_4_L has higher sensitivity for Cd^2+^ than other metallic ions that are based upon change in color by naked eyes. The mechanism of fluorescence change has been well demonstrated. The sensor H_4_L as a reliable fluorescence probe displayed high sensitivity toward Cd^2+^ in water-containing systems, and was able to detect Cd^2+^ in food samples.

## 2. Experimental

### 2.1. Materials and Methods

All of the reactions were performed under an air atmosphere. Boron tribromide (99.9%), methyl trioctyl ammonium chloride (90%), 2-hydroxy-3-methoxybenzaldehyde (99%), and pyridinium chlorochromate (98%) were gained from Alfa Aesar. Hydrobromic acid 33 wt % acetic acid solution was gained from J & K Scientific Ltd. (Beijing, China). Other solvents and reagents that were used in this work were analytical grade from Tianjin Chemical Reagent Factory (Tianjin, China). Melting points were measured using a microscopic melting point apparatus made in Beijing Taike Instrument Limited Company (Beijing, China) and were uncorrected. ^1^H NMR spectra were made via German Bruker AVANCEDRX-400 spectrophotometer (Karlsruhe, Germany). All of the UV–vis and fluorescence spectra tests were measured on a Shimadzu UV-2550 (Kyoto, Japan) and Perkin-Elmer LS-55 spectrometer (Waltham, MA, USA). The solvent also has a great influence on the fluorescence of the complex. Through a series of experiments, we finally obtained the best selectivity for cadmium ions in Tris-phosphate buffer (c = 0.2 M, DMF/H_2_O = 9:1, *v*/*v*, pH = 7.0) [59,60].

### 2.2. Preperation of Sensor H_4_L

**Scheme 1:**
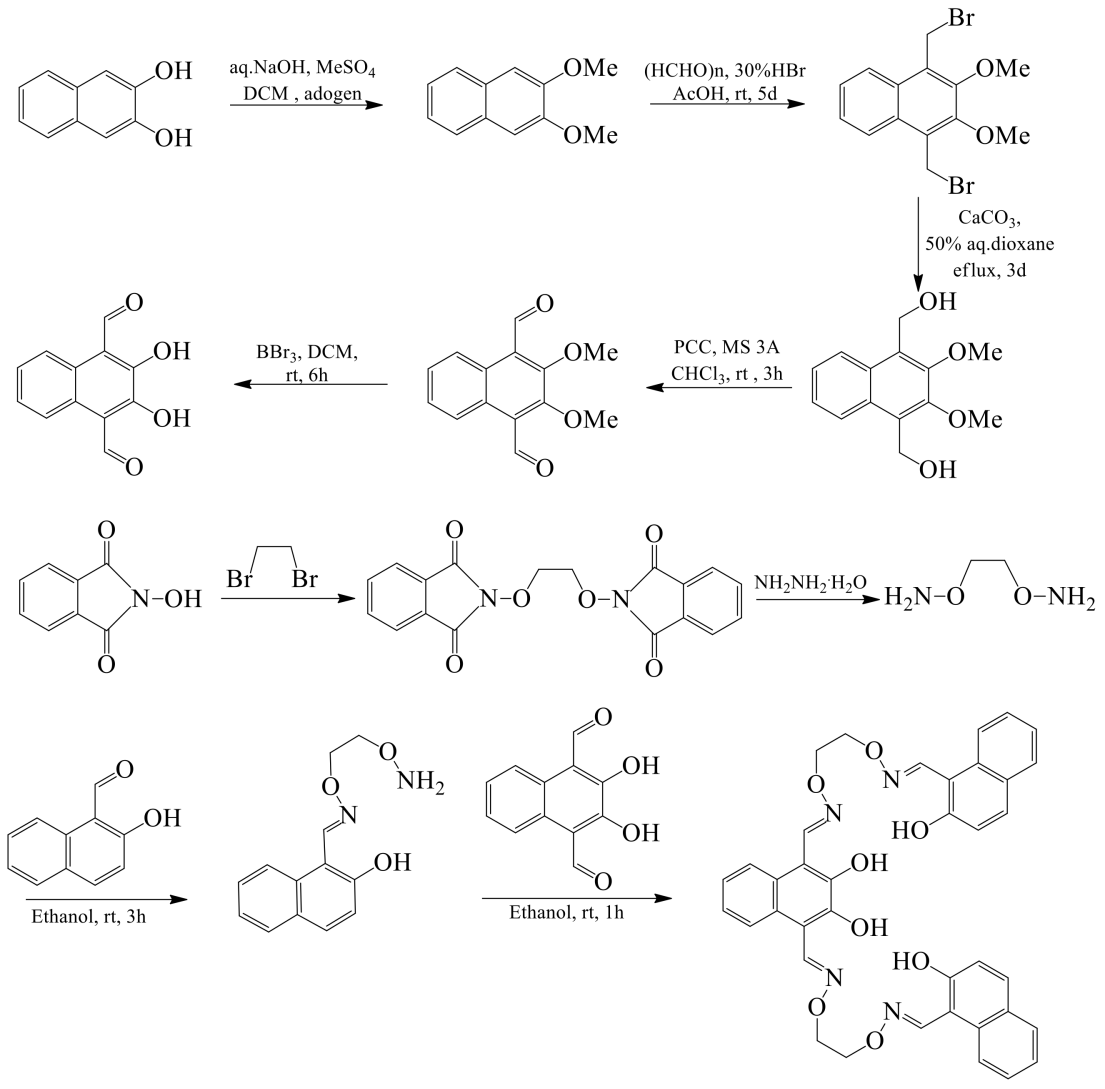
Synthetic route to sensor H_4_L.

**Figure 1:**
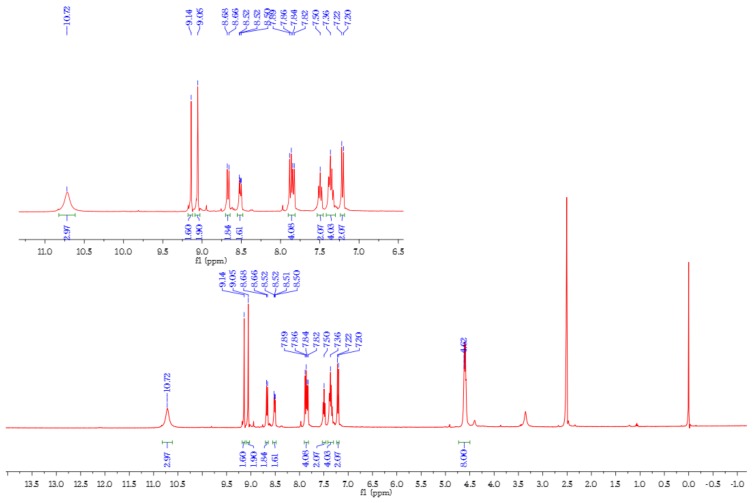
^1^HNMR spectra of sensor H_4_L in DMSO.

The bis(salamo)-type tetraoxime sensor H_4_L was prepared on the basis of the reported methods [61,62,63,64,65,66,67,68]. The ^1^H NMR, IR, and UV-vis spectra of H_4_L are nearly consistent with the previous result (Figure 1). The major reaction steps of sensor H_4_L are demonstrated in Scheme 1.

## 3. Results and Discussion

### 3.1. pH Effect of Sensor H_4_L

**Figure 2:**
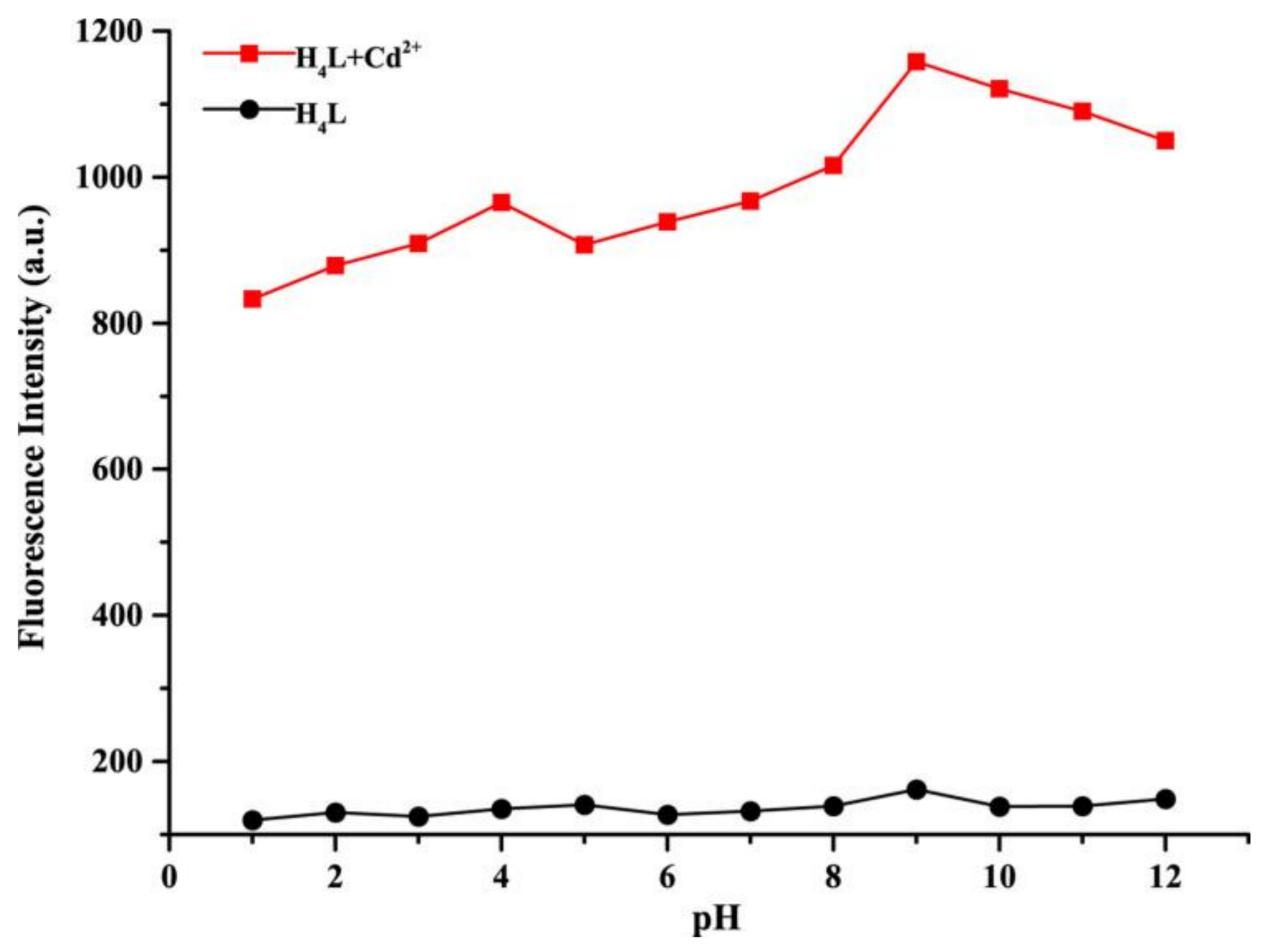
Changes in fluorescence intensity of H_4_L (c = 1 × 10^−5^ M) and Cd^2+^ (c = 1 × 10^−3^ M) performed at different pH values at room temperature. (DMF/H_2_O = 9:1, *v*/*v*, λ_ex_ = 323 nm, λ_e__m_ = 495 nm).

For the sake of avoid the disturbance by protons in the recognition process of heavy metallic ions and to get optimum conditions, we concentrated on the pH influence on the fluorescence intensity. As depicted in Figure 2, the sensor H_4_L have barely changed in the fluorescent intensity from pH = 3.0 to 11.0, indicating that sensor H_4_L was consistent. The weak fluorescent sensor H_4_L may be owing to intra-molecular photo-induced electron transfer. However, H_4_L-Cd^2+^ showed strong fluorescence, on account of the bonding of H_4_L with Cd^2+^ lead to the inhibition of intra-molecular photo-induced electron transfer process. The results indicated that sensor H_4_L can be used to detect Cd^2+^ and the process of detection was not affected greatly by pH values.

### 3.2. General UV–vis Measurements

**Figure 3:**
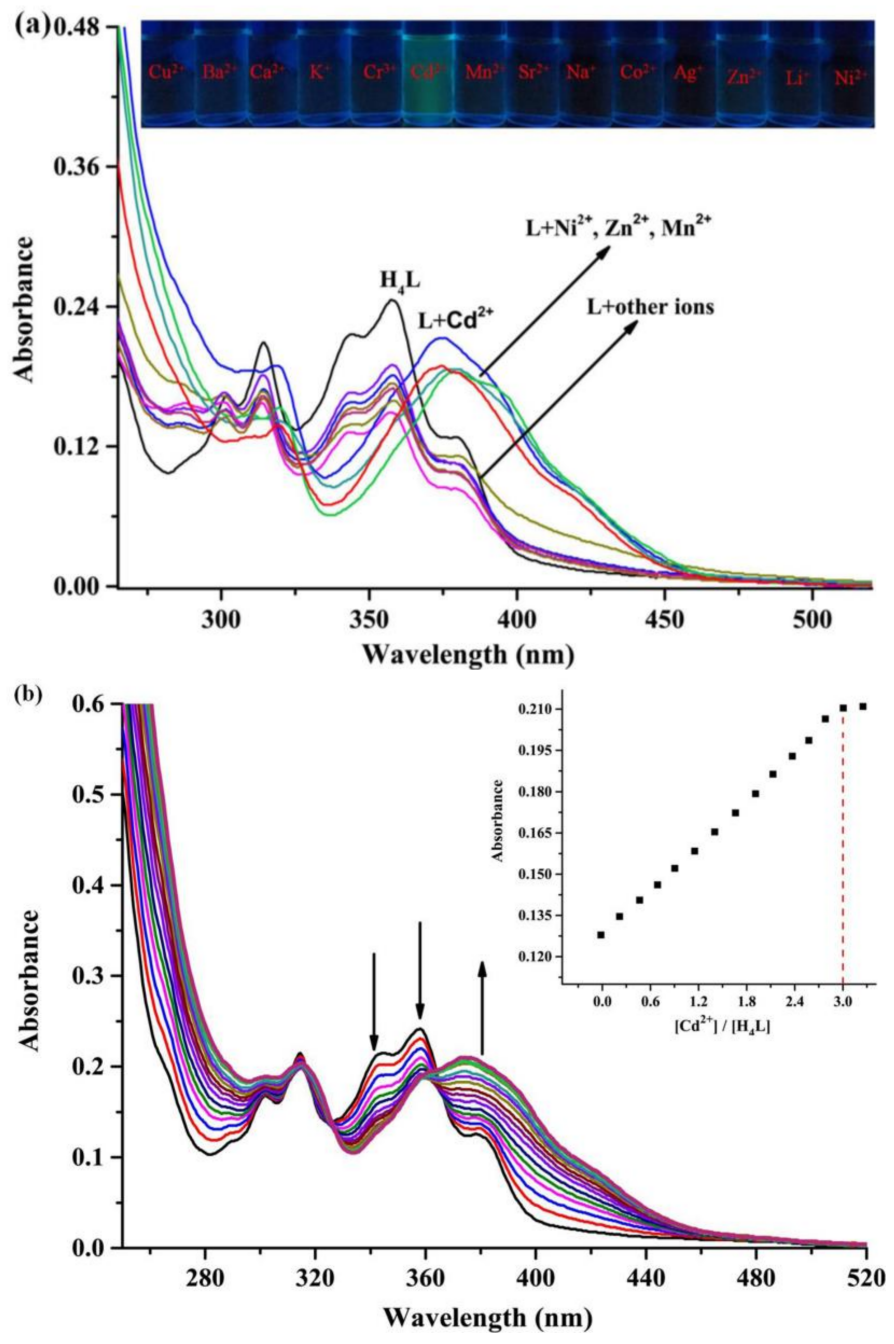
(**a**) UV-vis spectra of sensor H_4_L (1 × 10^−5^ M) recorded in Tris-phosphate buffer (c = 0.2 M, DMF/H_2_O = 9:1, *v*/*v*, pH = 7.0) solutions after addition of 3.0 equiv. of metallic ions (1 × 10^−3^ M); (**b**) Absorption spectra of sensor H_4_L in Tris-phosphate buffer (c = 0.2 M, DMF/H_2_O = 9:1, *v*/*v*, pH = 7.0) solutions upon gradual addition of Cd^2+^ (0.0 to 3.0 equiv.).

UV–vis spectra of sensor H_4_L in the existence of 14 metallic ions (Cd^2+^, Cu^2+^, Ba^2+^, Ca^2+^, K^+^, Cr^3+^, Mn^2+^, Sr^2+^, Co^2+^, Na^+^, Li^+^, Ni^2+^, Ag^+^, and Zn^2+^ with nitrate anions) were investigated. As shown in Figure 3a, sensor H_4_L have two intense absorption bands at 313 and 358 nm, which could be attributed to π → π* transition and reveals that sensor H_4_L includes a larger conjugation system. However, when 3.0 equiv. Cd^2+^ were added into the mixed solution, a new absorption peak emerged at 375 nm (Figure 3a). Meanwhile, the change in absorbance is almost same for Cd^2+^, Ni^2+^, Zn^2+^, and Mn^2+^ ions, indicating that the H_4_L were also involved in coordination to the Cd^2+^, Ni^2+^, Zn^2+^, and Mn^2+^ ions, respectively. However, when Cd^2+^ ion was added, sensor H_4_L could show a clear change in color from faint yellow to green that can be very easily observed via the naked eyes under UV light at 365 nm, while other metallic ions do not induce such a change. The colour change that is observed is mainly due to the charge transfer transition of the complex. The results revealed sensor H_4_L could be applied to selective identification of Cd^2+^ according to the color changes.

The responses of sensor H_4_L to Cd^2+^ (1 × 10^−3^ M) were studied further via UV–vis titration experiments, as depicted in Figure 3b. With the addition of Cd^2+^ from 0.0–3.0 equiv. in Tris-phosphate buffer (c = 0.2 M, DMF/H_2_O = 9:1, *v*/*v*, pH = 7.0) solutions, two peaks that were observed at 342 and 358 nm were clearly declined and a new band at 375 nm emerged. Meanwhile, one definitive isosbestic point was also noted at 326 nm, indicating that a new species is produced. When 3.0 equiv. Cd^2+^ was added, the absorption no longer change, which showed 1:3 stoichiometry between Cd^2+^ and sensor H_4_L (Figure 3b).

### 3.3. General Fluorescence Measurements

**Figure 4:**
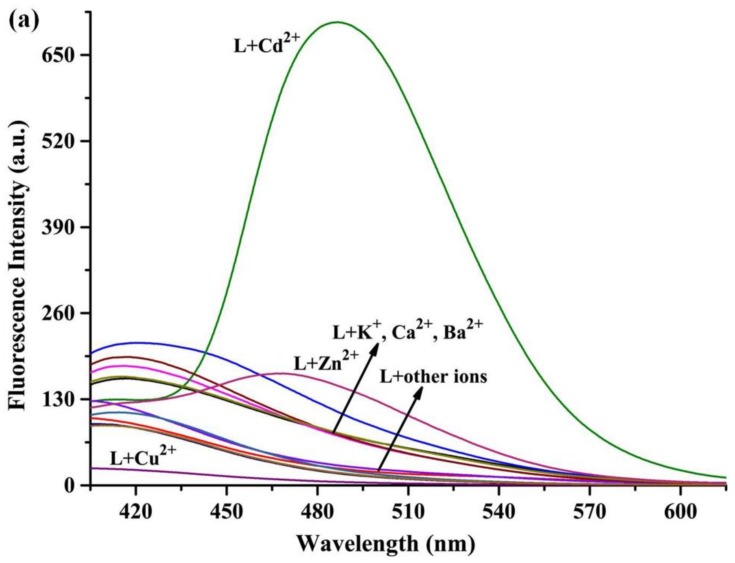

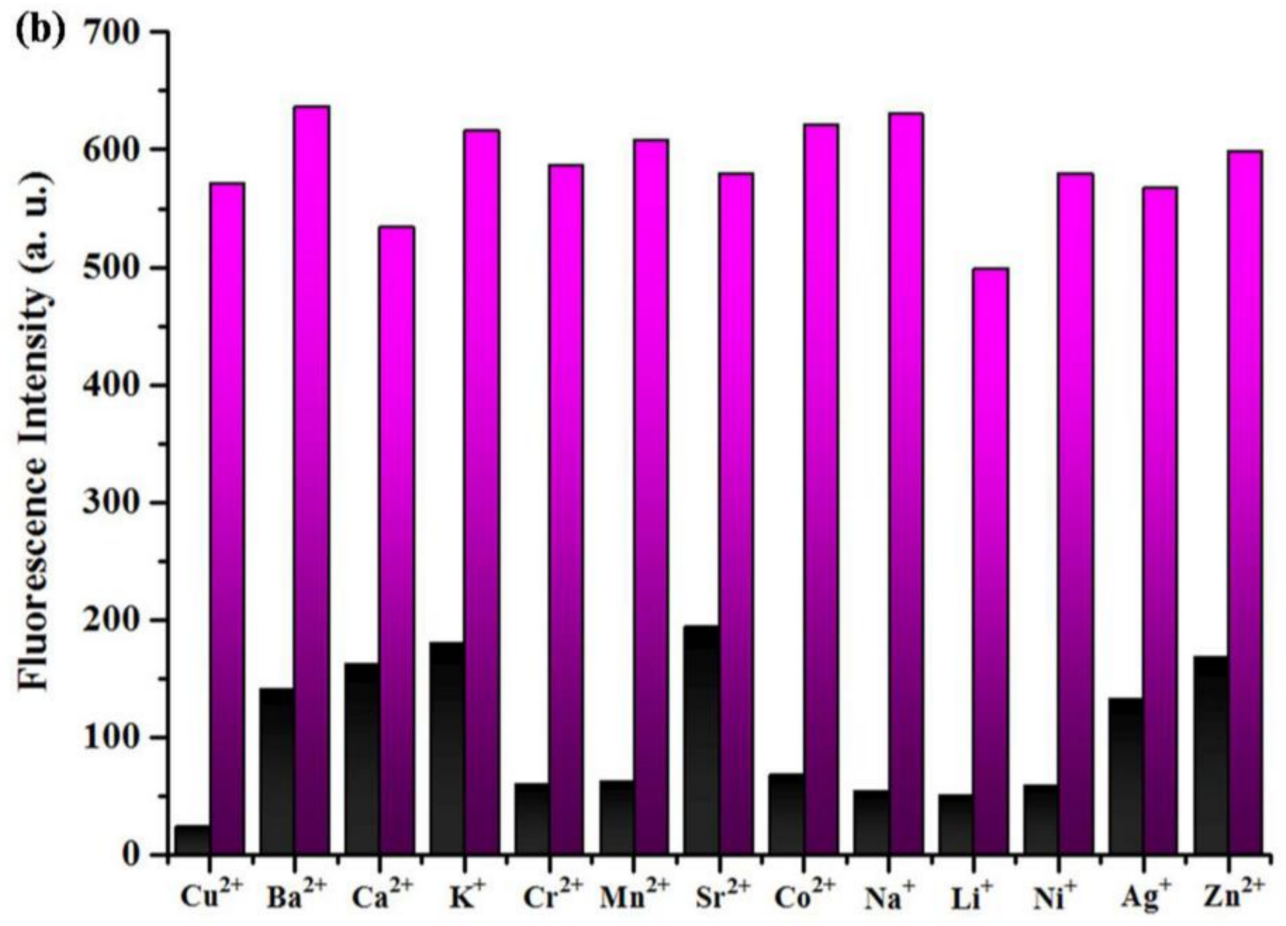
(**a**) Fluorescent spectra and (**b**) fluorescent intensity at 323 nm of sensor H_4_L (1 × 10^−5^ M) in the existence of different metallic ions (c = 1 × 10^−3^ M, excess amounts Cu^2+^, Ba^2+^, Ca^2+^, K^+^, Cr^3+^, Mn^2+^, Sr^2+^, Co^2+^, Na^+^, Li^+^, Ni^2+^, Ag^+^, and Zn^2+^) in Tris-phosphate buffer (c = 0.2 M, DMF/H_2_O = 9:1, *v*/*v*, pH = 7.0, λ_ex_ = 323 nm) solution (the black bars delegate the addition of excess amounts of metallic ions to the solution of probe H_4_L and the pink bars delegate the subsequent addition of Cd^2+^ to the mixed solution).

For the sake of evaluating the selectivity of sensor H_4_L, original screening of H_4_L to the bonding ability of metallic ions was performed in Tris-phosphate buffer (c = 0.2 M, DMF/H_2_O = 9:1, *v*/*v*, pH = 7.0) solutions. The fluorescent spectra of sensor H_4_L in the existence of a series of metallic ions (Cd^2+^, Cu^2+^, Ba^2+^, Ca^2+^, K^+^, Cr^3+^, Mn^2+^, Sr^2+^, Co^2+^, Na^+^, Li^+^, Ni^2+^, Ag^+^, and Zn^2+^ with nitrate anions) were gained, followed via excitation at 323 nm. As shown in Figure 4a, when Cd^2+^ ion was added, the position of the emission peak was red-shifted from 412 to 486 nm, the fluorescence intensity increased from 125 to 712, and the fluorescence quantum yield increased from 2.6 to 13.8%. Significantly, H_4_L showed excellent selectivity for Cd^2+^ in Tris-phosphate buffer (c = 0.2 M, DMF/H_2_O = 9:1, *v*/*v*, pH = 7.0) solutions with a strong fluorescence response, with either a very weak or no fluorescence response to other metallic ions (Figure 4a). It is different from the previously reported sensors for Cd^2+^ showing a wide range of response to Zn^2+^, sensor H_4_L nearly non-fluorescent respond to Zn^2+^ could be owing to its rigid cavity with bigger size. Therefore, this rigid cavity could be definitely suitable for bonding with Cd^2+^, but not suitable for bonding with Zn^2+^ that possess smaller ionic radius. Furthermore, according to the corrected Benesi-Hildebrand formula, the bonding constant for the bonding of Cd^2+^ to sensor H_4_L was estimated as 4.98 × 10^4^ M^−1^ and Zn^2+^ to sensor H_4_L was estimated as 3.89 × 10^4^ M^−1^ [69]. The result suggested that sensor H_4_L displays outstanding selectivity for Cd^2+^ than other metallic ions.

For the sake of the practicability of sensor H_4_L as a receptor of Cd^2+^ selective probe, competitive experiments were performed with various metallic ions in Tris-phosphate buffer (c = 0.2 M, DMF/H_2_O = 9:1, *v*/*v*, pH = 7.0) solutions. As shown in Figure 4b, there are no other ions can result any clear changes in the fluorescent spectrum of sensor. The selectivity of sensor H_4_L for Cd^2+^ and other metallic ions was measured. These results also demonstrated that other metallic ions could not interfere with the detection of Cd^2+^.

**Figure 5:**
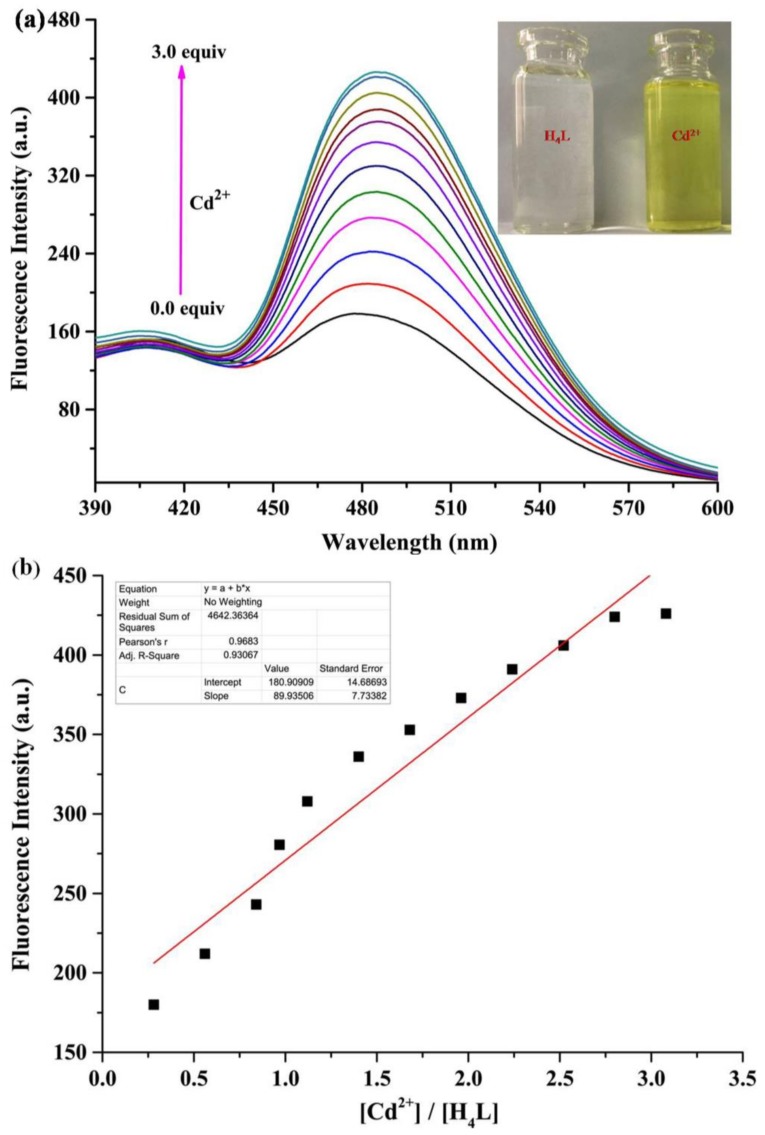
(**a**) Fluorescent emission spectra of sensor H_4_L (1 × 10^−5^ M) with subsequent addition of Cd^2+^ (0–3.0 equiv. λex = 323 nm) in Tris-phosphate buffer (c = 0.2 M, DMF/H_2_O = 9:1, *v*/*v*, pH = 7.0) solutions. Inset: Naked-eyes visible colour changes of sensor H_4_L solution before and after addition of Cd^2+^. (**b**) Linear fitting of sensor H_4_L to Cd^2+^ bonding constant.

To further understand the coordination of sensor H_4_L with the Cd^2+^, fluorescent responses of sensor H_4_L to changing concentrations of Cd^2+^ in Tris-phosphate buffer (c = 0.2 M, DMF/H_2_O = 9:1, *v*/*v*, pH = 7.0) solutions at room temperature were investigated in Figure 5. With the addition of increasing Cd^2+^ at an excitation wavelength of 323 nm, the fluorescent emission intensity at 485 nm gradually raised while the intensity at 407 nm reduced. Furthermore, after the addition of Cd^2+^, the absorbance at 485 nm exhibited a sharp increase when the ratio of [Cd^2+^]/[H_4_L] is below 3:1, and no longer change when the ratio reaches 3:1. In addition, the fitting curve of fluorescence emission intensity with Cd^2+^ concentrations was obtained by the data obtained of the fluorescence titration experiment (Figure 5). We have performed ^1^H NMR spectra of sensor H_4_L and in which the presence of 3.0 equiv. Cd^2+^ (Figure S1). Moreover, the detection limit is a considerably significant parameter in molecular recognition, the LOD and LOQ parameters were estimated to be 8.61 × 10^−7^ M and 2.87 × 10^−6^ M, respectively [61,69]. The LOD and LOQ were calculated based on the following equations:

LOD = 3 × δ/S; LOQ = 10 × δ/S.

where S represents the standard deviation of the blank measurements, and δ is the slope of the intensity versus sample concentration curve.

At the same time, the Hill equation is used in determining the binding constant of ions and H_4_L, the bonding constant for the bonding of Cd^2+^ to sensor H_4_L was estimated as 4.98 × 10^4^ M^−1^ [48,59]. These results indicate that probe H_4_L displays satisfactory Cd^2+^ detecting ability.

log(F-F_min_)/(F_max_-F) = log *K* + n log [Cd^2+^]; n = 3.

where F_min_, F_max_, and F are the emission intensities in the absence, presence of saturated Cd^2+^, and the addition of a given amount of Cd^2+^ concentration, respectively. [Cd^2+^] is the concentration of free Cd^2+^.

**Figure 6:**
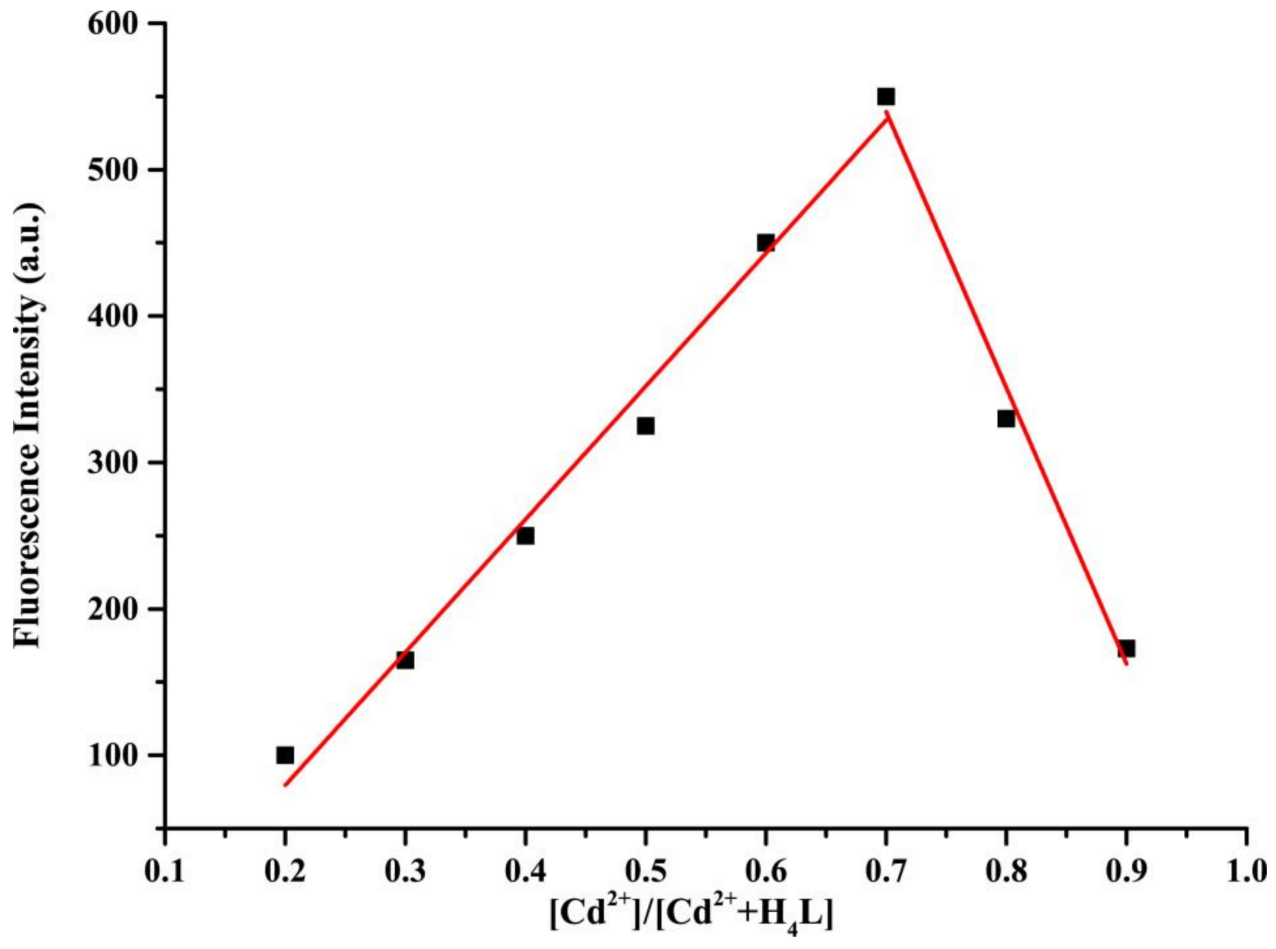
The job’s plot examined between Cd^2+^ and H_4_L, indicating the 3:1 stoichiometry, which was carried out by fluorescence spectra (λex = 323 nm).

To know the stoichiometry between the sensor H_4_L and Cd^2+^ in Tris-phosphate buffer (c = 0.2 M, DMF/H_2_O = 9:1, *v*/*v*, pH = 7.0) solutions, job’s plot has been performed (Figure 6). When the molar fraction of Cd^2+^ was 0.75, the intensity at 495 nm reached an extreme value, indicating the formation of a 3:1 complex between Cd^2+^ and H_4_L.

**Figure 7:**
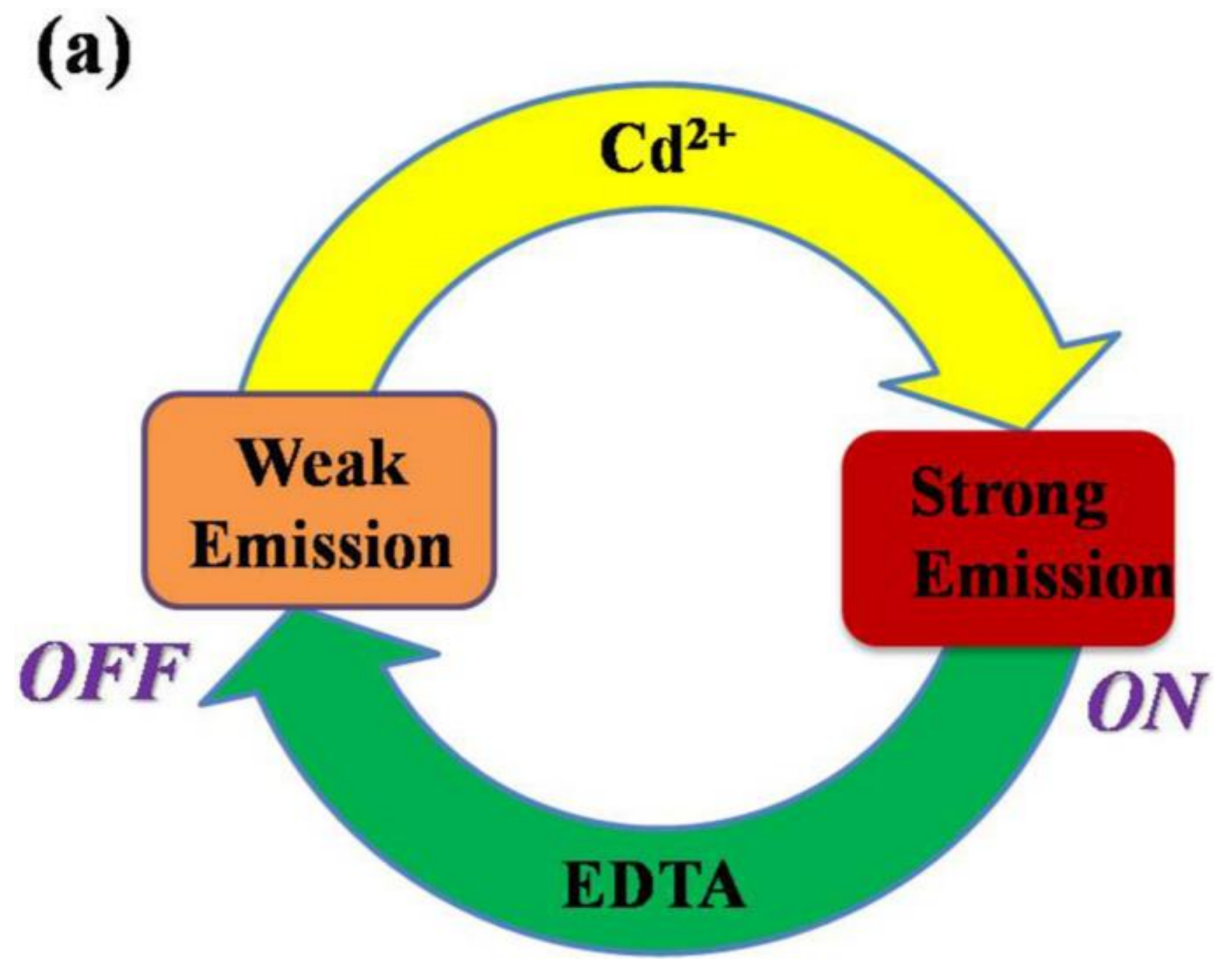

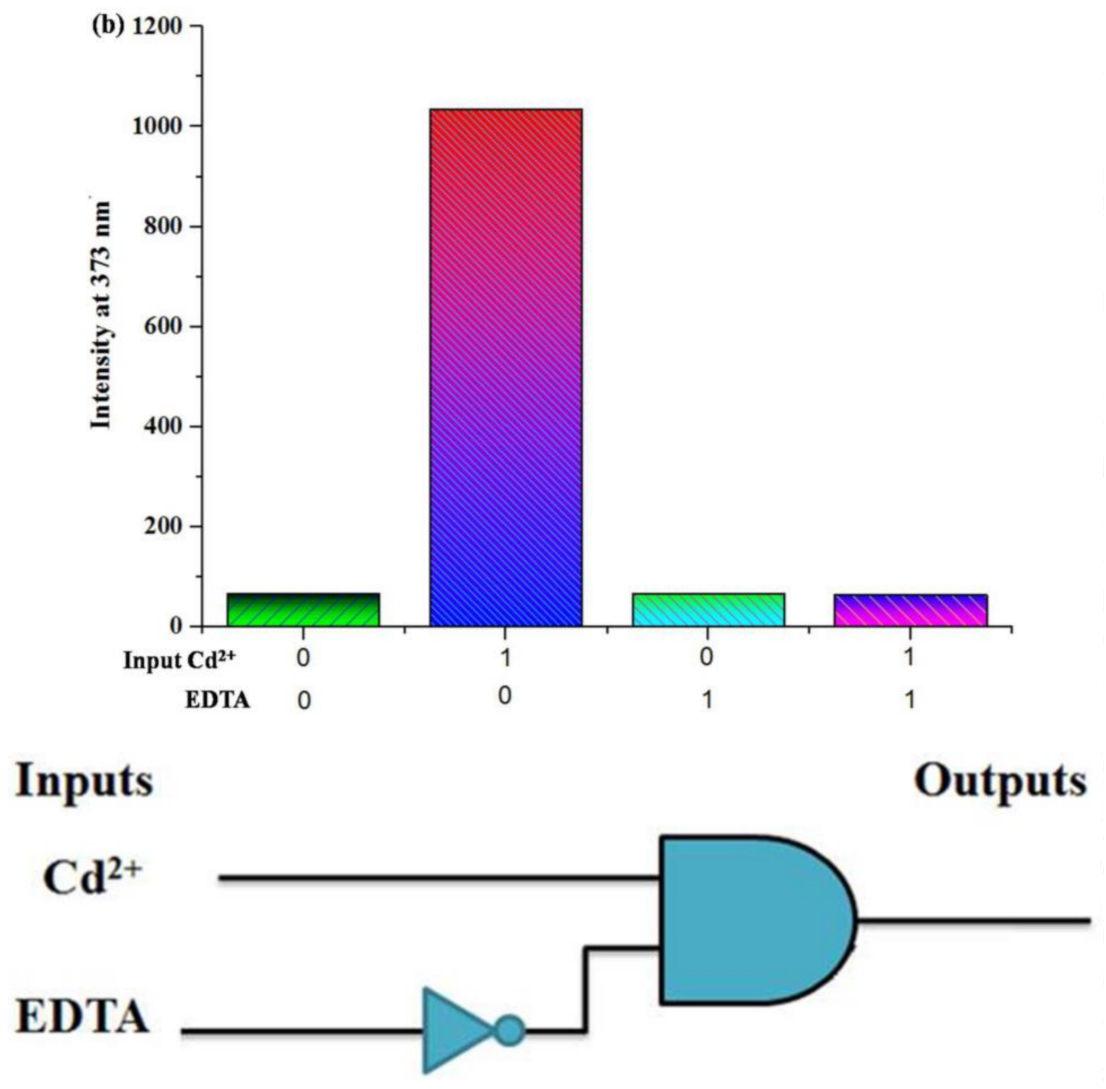
(**a**) Schematic presentation of “OFF–ON” system for sensor H_4_L (c = 1 × 10^−5^ M) in the existence of Cd^2+^ (c = 1 × 10^−3^ M) and EDTA (c = 1 × 10^−3^ M); (**b**) Performance of sensor H_4_L in the INHIBIT gate mode in Tris-phosphate buffer (c = 0.2 M, DMF/H_2_O = 9:1, *v*/*v*, pH = 7.0, λex = 373 nm). The bars display the fluorescent output of sensor H_4_L at 373 nm in the existence of Cd^2+^ and EDTA serve as inputs.

Furthermore, according to the reversible fluorescent switch of the sensor H_4_L to Cd^2+^ in a coordination compound solution, we regard it as a two-input molecular logic gate, while the emission at 373 nm serves as the output. As depicted in Figure 7 and Table 1, when the output was zero (both the Cd^2+^ and EDTA are absent), corresponding the gate being closed and this system shows weak fluorescence. When Cd^2+^ alone was existent, the output is one and the relevant to the gate being open, so intense fluorescence was observed. Thus, the sensor H_4_L is able to serve as a logic gate. This result demonstrates that the sensor H_4_L as a reversible fluorescence probe.

### 3.4. The Detecting Mechanism of H_4_L for Cd^2+^

**Figure 8:**
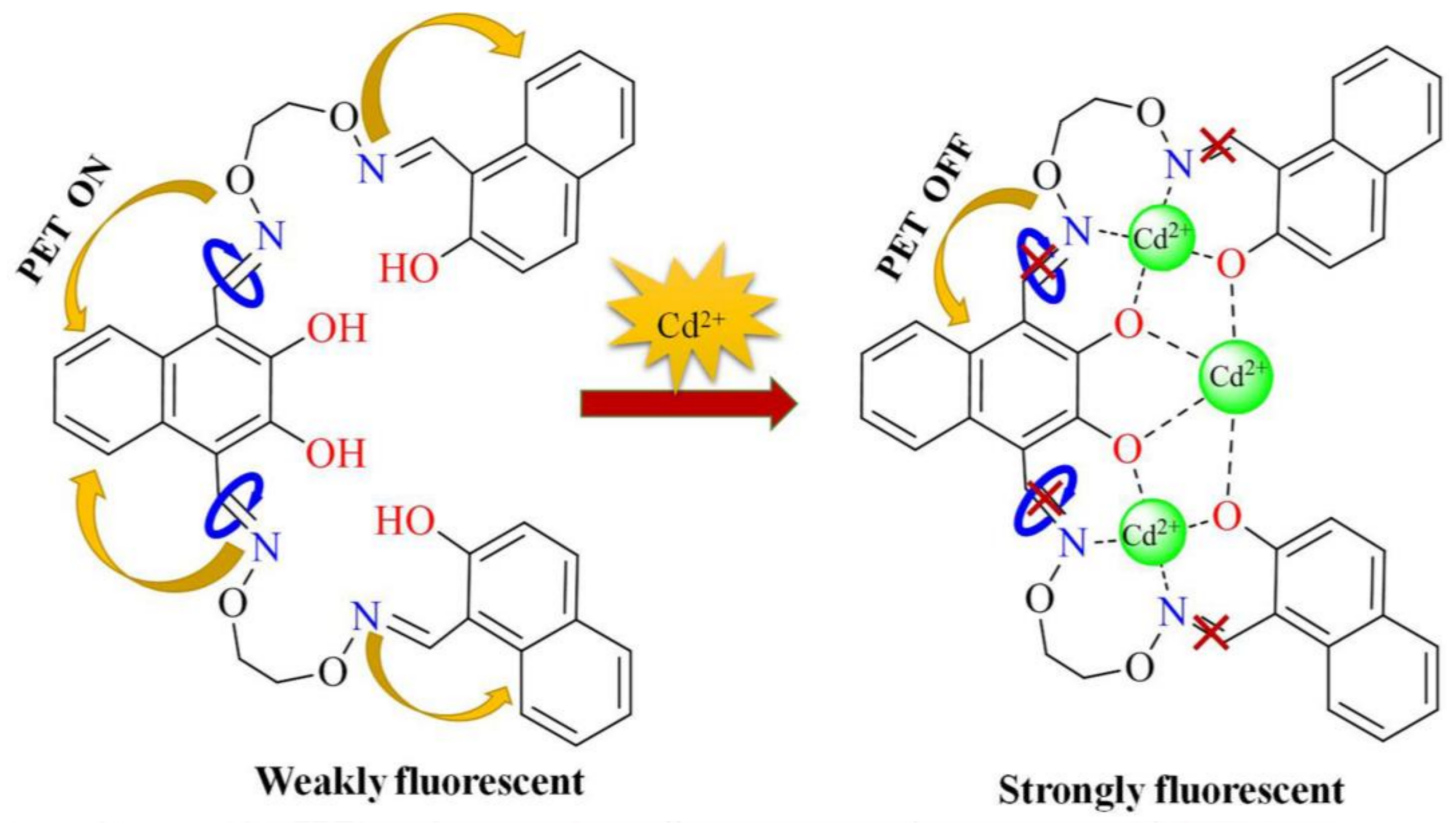
The sensing mechanism of the sensor H_4_L for Cd^2+^.

According to the fluorescent spectra, the detecting mechanism of the sensor H_4_L for Cd^2+^ was suggested, as follows (Figure 8). The fluorescent intensity of the sensor H_4_L response to Cd^2+^ may be assigned to CHEF and PET. Before being coordinated with Cd^2+^, sensor H_4_L displayed a weaker fluorescence due to the lone pair of electrons of nitrogen atoms, which gives rise to an intra-molecular PET. Furthermore, the lone electron pairs of the nitrogen atoms give rise to a nonradiative process by the n-π* state, which also led to a wide degree of fluorescent quenching. Conversely, after H_4_L was coordinated to Cd^2+^, the radiation process was primarily via the π-π* state and the coordination compound was more rigid [48,59]. In addition, the PET process was restrained by the addition of Cd^2+^ analyte at the receptor site. It is obvious that the appearance of the ICT process influenced PET in this system, but fluorophore and receptor distances and the orientation between them can also contribute in the overall PET process.

### 3.5. Time and Temperature Effect of Probe H_4_L

**Figure 9:**
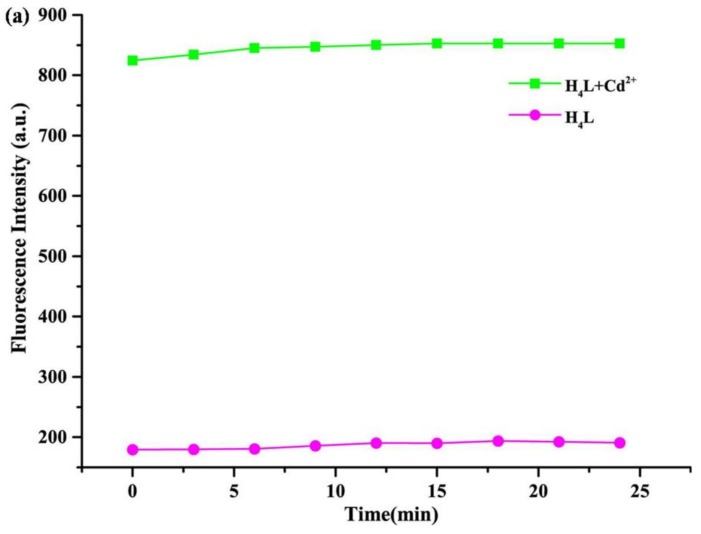

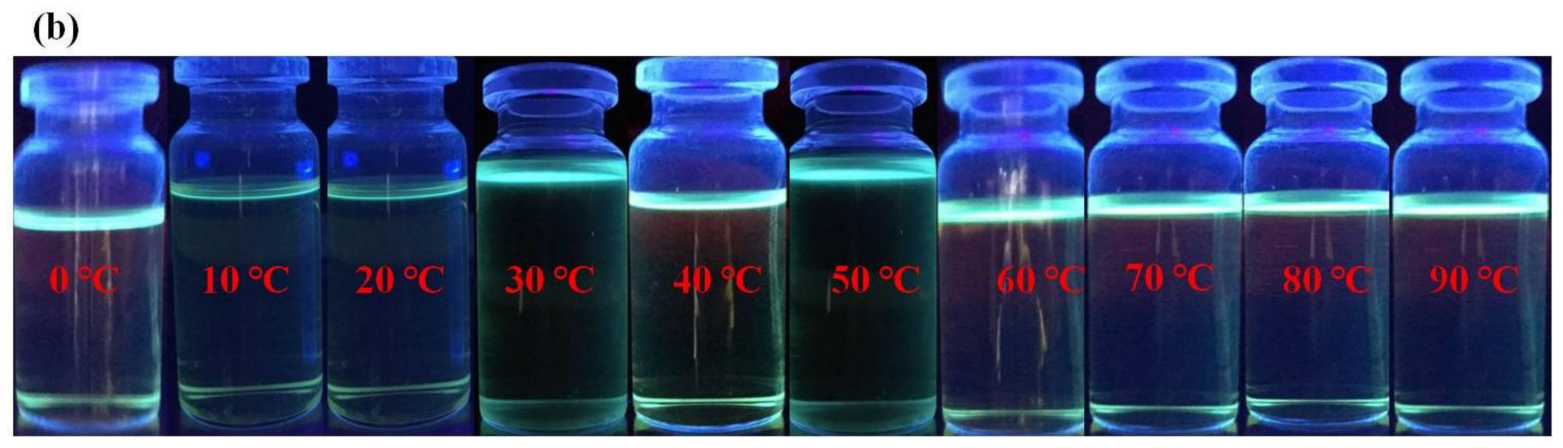
(**a**) Fluorescence intensity changes of the solution contain sensor H_4_L and 3.0 equiv. Cd^2+^ with the delay of time; (**b**) Fluorescent intensity changes of the solution contain H_4_L and 3.0 equiv. Cd^2+^ at different temperatures.

The influences of time and temperature on the fluorescent intensity of sensor H_4_L toward Cd^2+^ were also studied in Tris-phosphate buffer (c = 0.2 M, DMF/H_2_O = 9:1, *v*/*v*, pH = 7.0) solutions. Figure 9 illustrates that fluorescence intensity of sensor H_4_L did not vary with further prolong the reaction time. Besides, the fluorescent intensity of sensor H_4_L almost constant in the temperature range of 0–90 °C. Therefore, sensor H_4_L could be used for rapid response to Cd^2+^ in room temperature, which is of significant practicability for the detection of Cd^2+^.

### 3.6. Test Strips Measurements

**Figure 10:**
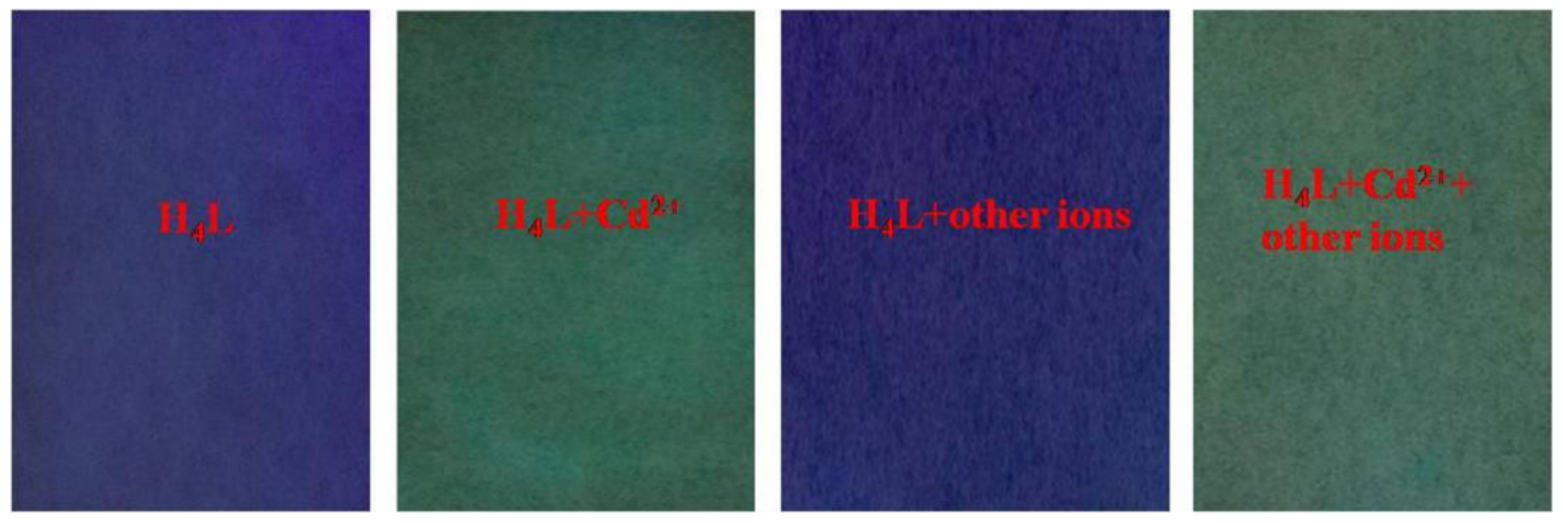
The result of colorimetric measured photographs with sensor H_4_L for detecting Cd^2+^ under irradiation at 365 nm.

Currently, test strips analytical equipment have gained momentous attention on account of their high sensitivity, low-cost, and quick response. It is perhaps the most convenient of modern detection tools, as a change in color could be seen via the naked eyes. Hence, in this study, filter papers were performed via soaking filter papers into Tris-phosphate buffer (c = 0.2 M, DMF/H_2_O = 9:1, *v*/*v*, pH = 7.0) mixed solutions of probe H_4_L (1 × 10^−5^ M), and then drying by exposure to air. The filter papers, including sensor H_4_L, were applied to detect Cd^2+^ and other metallic ions. As depicted in Figure 10, after Cd^2+^ and the other metallic ions were added on the test tools, respectively, the distinct color changes were seen merely with Cd^2+^ solution under UV light at 365 nm, and potentially competitive metallic ions have no work on the detection of Cd^2+^ via the filter papers (Figure S2). It is a really actual convenient method that quickly measures the Cd^2+^, and it could be able to be satisfactorily used in the fields of food security and environmental surveillance.

### 3.7. Application in Food Samples

**Figure 11:**
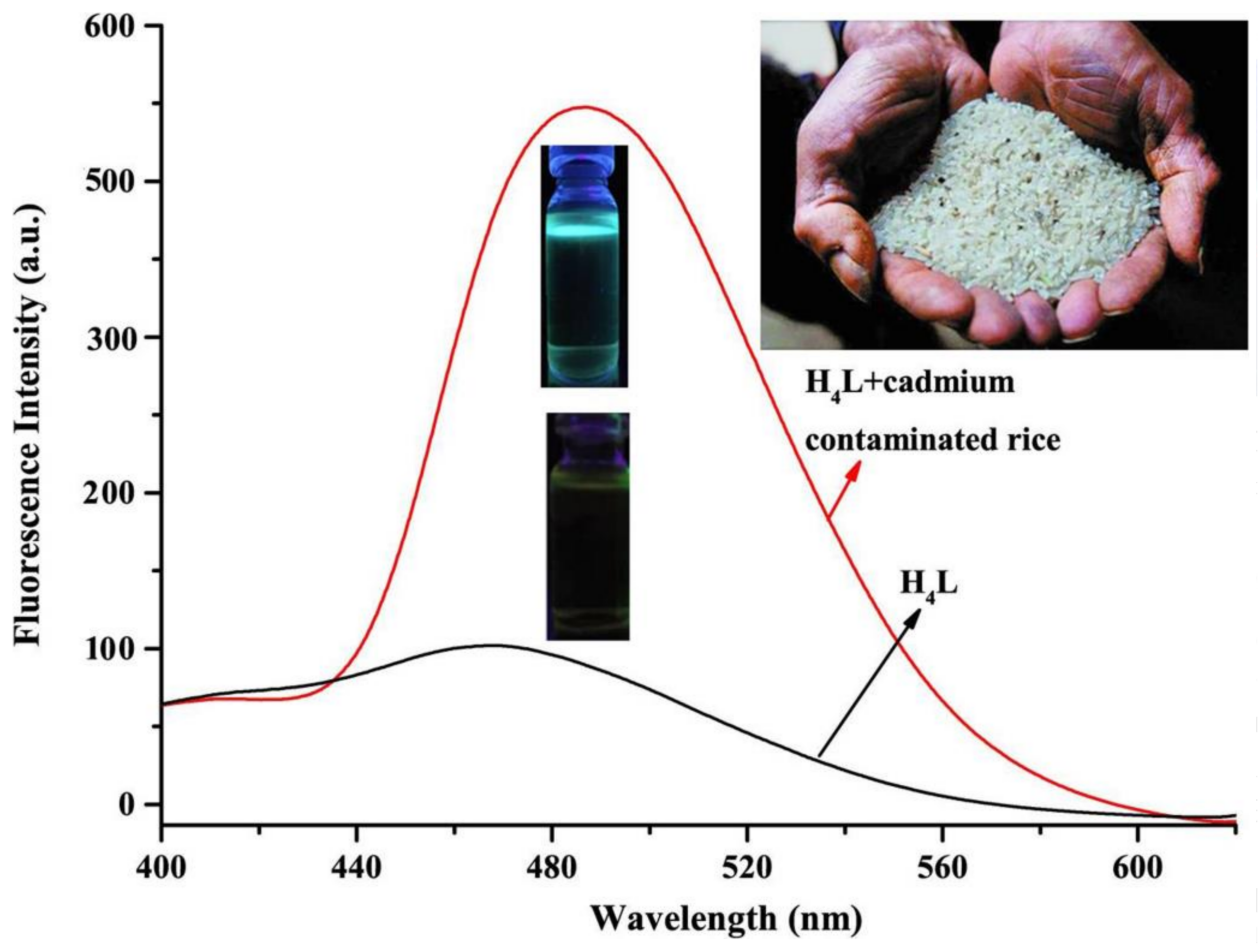
Fluorescence spectral response of sensor H_4_L in cadmium contaminated rice.

Furthermore, we investigated the applicability of sensor H_4_L in food samples. 500 mg of crushed naturally containing cadmium rice were put into the PTFE microwave digestion tank by the addition of 3 mL of HNO_3_ and 4 mL of H_2_O_2_, and the mixture solution containing cadmium(II) needs be digested after keeping 30–60 min. Then, the mixture solution was dried in vacuo and was added into the mixed solution of DMF and H_2_O (c = 1 × 10^−3^ M, DMF/H_2_O = 9:1, *v*/*v*, pH = 7). As shown in Figure 11, after added the sample solution to sensor H_4_L, the fluorescence intensity is on the increase.

## 4. Conclusions

In this paper, we presented a new bis(salamo)-type fluorescence sensor H_4_L, which could serve as a great promising analytical kit for measuring Cd^2+^ via different fluorescence changes and changes in color from light yellow to green that can be measured via naked eyes. The detection limit about fluorescent response of probe H_4_L to Cd^2+^ was down to 8.61 × 10^−7^ M. As designed, the filter papers could conveniently, efficiently, and simply detect Cd^2+^ in solution. Moreover, Cd^2+^ in the food samples was detected by the sensor H_4_L simply and effectively. We believe this study will inspire the development of bis(salamo)-based chemosensor by optimizing unsaturated metal coordinating sites for practicability to many other metallic ions detecting in analytical chemistry, medical treatment, biological, and environmental fields.

## Figures and Tables

**Table 1:** The molecular logic gate and the each symbolic expression of the INHIBIT logic gate function.

Inputs		Outputs	
Cd^2+^	EDTA	(INHIBIT Logic Gate) Intensity λ_max_	
0	0	low flu.	0
1	0	high flu.	1
0	1	low flu.	0
1	1	low flu.	0

## Supplementary Materials

The following are available online at http://www.mdpi.com/1996-1944/11/4/523/s1, Figure S1: ^1^H NMR titration in upon addition of 3.0 equiv. Cd^2+^, Figure S2: The result of colorimetric measured photographs with sensor H_4_L for detecting Cd^2+^ under irradiation at 365 nm.

## References

[1] Chai L.Q., Tang L.J., Chen L.C., Huang J.J. (2017). Structural, spectral, electrochemical and DFT studies of two mononuclear manganese(II) and zinc(II) complexes. Polyhedron.

[2] Chai L.Q., Zhang K.Y., Tang L.J., Zhang J.Y., Zhang H.S. (2017). Two mono- and dinuclear Ni(II) complexes constructed from quinazoline-type ligands: Synthesis, X-ray structures, spectroscopic, electrochemical, thermal, and antimicrobial studies. Polyhedron.

[3] Chai L.Q., Zhang J.Y., Chen L.C., Li Y.X., Tang L.J. (2016). Synthesis, crystal structure, spectroscopic properties and DFT calculations of a new schiff base-type zinc(II) complex. Res. Chem. Intermed..

[4] Chai L.Q., Li Y.X., Chen L.C., Zhang J.Y., Huang J.J. (2016). Synthesis, X-ray structure, spectroscopic, electrochemical properties and DFT calculation of a bridged dinuclear copper(II) complex. Inorg. Chim. Acta.

[5] Liu X., Wang P., Fu J., Yao K., Xue K., Xu K. (2017). Turn-on fluorescent sensor for zinc and cadmium ions based on quinolone and its sequential response to phosphate. J. Lumin..

[6] Marettová E., Maretta M., Legáth J. (2015). Toxic effects of cadmium on testis of birds and mammals: A review. Anim. Reprod. Sci..

[7] Al-Khaldi F.A., Abusharkh B., Khaled M., Atieh M.A., Nasser M.S. (2015). Adsorptive removal of cadmium(II) ions from liquid phase using acid modified carbon-based adsorbents. J. Mol. Liq..

[8] Akesson A., Julin B., Wolk A. (2008). Long-term dietary cadmium intake and postmenopausal endometrial cancer incidence: A population-based prospective cohort study. Cancer Res..

[9] Yang L.L., Liu X.M., Liu K., Liu H., Zhao F.Y., Ruan W.J. (2014). A polypyridyl-pyrene based off-on Cd^2+^ fluorescent sensor for aqueous phase analysis and living cell imaging. Talanta.

[10] Vinod K., Gupta M.R., Ganjali P., Norouzi H., Khani A.N., Shilpi A. (2011). Electrochemical analysis of some toxic metals by ion–selective electrodes. Crit. Rev. Anal. Chem..

[11] Gupta V.K., Jain A.K., Agarwal S., Maheshwari G. (2007). An iron(III) ion-selective sensor based on a mu-bis(tridentate) ligand. Talanta.

[12] Gupta V.K., Chandra S., Lang H. (2005). A highly selective mercury electrode based on a diamine donor ligand. Talanta.

[13] Gupta V.K., Sethi B., Sharma R.A., Agarwal S., Bharti A. (2013). Mercury selective potentiometric sensor based on low rim functionalized thiacalix-arene as a cationic receptor. J. Mol. Liq..

[14] Xu Z., Zhang L., Guo R., Xiang T., Wu C., Zheng Z. (2011). A highly sensitive and selective colorimetric and off–on fluorescent chemosensor for Cu^2+^, based on rhodamine derivative. Sens. Actuators B.

[15] Jun M.E., Roy B., Ahn K.H. (2011). “Turn-On” fluorescent sensing with “Reactive” probes. Chem. Commun..

[16] Du J., Hu M., Fan J., Peng X. (2012). Cheminform abstract: Fuorescent chemodosimeters using “mild” chemical events for the detection of small anions and cations in biological and environmental media. Chem. Soc. Rev..

[17] Chen X., Pradhan T., Wang F., Kim J.S., Yoon J. (2012). Fluorescent chemosensors based on spiroring-opening of xanthenes and related derivatives. Chem. Rev..

[18] Li X.Y., Chen L., Gao L., Zhang Y., Akogun S.F., Dong W.K. (2017). Syntheses, crystal structures and catalytic activities of two solvent-induced homotrinuclear Co(II) complexes with a naphthalenediol-based bis(Salamo)-type tetraoxime ligand. RSC Adv..

[19] Wang L., Ma J.C., Dong W.K., Zhu L.C., Zhang Y. (2016). A novel Self–assembled nickel(II)–cerium(III) heterotetranuclear dimer constructed from N_2_O_2_-type bisoxime and terephthalic acid: Synthesis, structure and photophysical properties. Z. Anorg. Allg. Chem..

[20] Ma J.C., Dong X.Y., Dong W.K., Zhang Y., Zhu L.C., Zhang J.T. (2016). An unexpected dinuclear Cu(II) complex with a bis(Salamo) chelating ligand: Synthesis, crystal structure, and photophysical properties. J. Coord. Chem..

[21] Tao C.H., Ma J.C., Zhu L.C., Zhang Y., Dong W.K. (2017). Heterobimetallic 3d–4f Zn(II)–Ln(III) (Ln = Sm, Eu, Tb and Dy) complexes with a N_2_O_4_ bisoxime chelate ligand and a simple auxiliary ligand Py: Syntheses, structures and luminescence properties. Polyhedron.

[22] Dong Y.J., Dong X.Y., Dong W.K., Zhang Y., Zhang L.S. (2017). Three asymmetric Salamo-type copper(II) and cobalt(II) complexes: Syntheses, structures, fluorescent properties. Polyhedron.

[23] Dong Y.J., Ma J.C., Zhu L.C., Dong W.K., Zhang Y. (2017). Four 3d–4f heteromultinuclear zinc(II)–lanthanide(III) complexes constructed from a distinct hexadentate N_2_O_2_-type ligand: Syntheses, structures and photophysical properties. J. Coord. Chem..

[24] Song X.Q., Liu P.P., Liu Y.A., Zhou J.J., Wang X.L. (2016). Two dodecanuclear heterometallic [Zn_6_Ln_6_] clusters constructed by a multidentate salicylamide salen-like ligand: Synthesis, structure, luminescence and magnetic properties. Dalton Trans..

[25] Liu P.P., Sheng L., Song X.Q., Xu W.Y., Liu Y.A. (2015). Synthesis, structure and magnetic properties of a new one dimensional manganese coordination polymer constructed by a new asymmetrical ligand. Inorg. Chim. Acta.

[26] Song X.Q., Liu P.P., Xiao Z.R., Li X., Liu Y.A. (2015). Four polynuclear complexes based on a versatile salicylamide salen-like ligand: Synthesis, structural variations and magnetic properties. Inorg. Chim. Acta.

[27] Song X.Q., Peng Y.J., Chen G.Q., Wang X.R., Liu P.P., Xu W.Y. (2015). Substituted group-directed assembly of Zn(II) coordination complexes based on two new structural related pyrazolone based Salen ligands: Syntheses, structures and fluorescence properties. Inorg. Chim. Acta.

[28] Liu P.P., Wang C.Y., Zhang M., Song X.Q. (2017). Pentanuclear sandwich-type Zn^II^-Ln^III^ clusters based on a new Salen-like salicylamide ligand: Structure, near-infrared emission and magnetic properties. Polyhedron.

[29] Wang P., Zhao L. (2015). Synthesis, structure and spectroscopic properties of the trinuclear cobalt(II) and nickel(II) complexes based on 2-hydroxynaphthaldehyde and bis(aminooxy)alkane. Spectrochim. Acta Part A.

[30] Wang P., Zhao L. (2016). An infinite 2D supramolecular cobalt(II) complex based on an asymmetric Salamo-type ligand: Synthesis, crystal structure, and spectral properties. Synth. React. Inorg. Met-Org. Nano-MetChem..

[31] Hu J.H., Li J.B., Qi J., Sun Y. (2015). Selective colorimetric and “turn-on” fluorimetric detection of cyanide using an acylhydrazone sensor in aqueous media. New J. Chem..

[32] Hu J.H., Li J.B., Qi J., Chen J.J. (2015). Highly selective and effective mercury(II) fluorescent sensor. New J. Chem..

[33] Li J.B., Hu J.H., Chen J.J., Qi J. (2014). Cyanide detection using a benzimidazole derivative in aqueous media. Spectrochim. Acta A.

[34] Hu J.H., Li J.B., Qi J., Sun Y. (2015). Acylhydrazone based fluorescent chemosensor for zinc in aqueous solution with high selectivity and sensitivity. Sens. Actuators B Chem..

[35] Sun Y., Hu J.H., Qi J., Li J.B. (2016). A highly selective colorimetric and “turn-on” fluorimetric chemosensor for detecting CN—Based on unsymmetrical azine derivatives in aqueous media. Spectrochim. Acta A.

[36] Hu J.H., Chen J.J., Li J.B., Qi J. (2014). A cyanide ion probe based on azosalicylic aldehyde of benzoyl hydrazone. Chin. J. Inorg. Chem..

[37] Qi J., Hu J.H., Chen J.J., Sun Y., Li J.B. (2016). Cyanide Detection using Azo-acylhydrazone in Aqueous Media with High Sensitivity and Selectivity. Curr. Anal. Chem..

[38] Hu J.H., Li J.B., Qi J., Sun Y. (2016). Studies on the crystal structure and characterization of *n*-(4-acetylphenyl)-na-(2-nitrobenzoyl)-thiourea. Phosphorus Sulfur Silicon Relat. Elem..

[39] Hu J.H., Sun Y., Qi J., Pei P.X., Lin Q., Zhang Y.M. (2016). A colorimetric and “turn-on” fluorimetric chemosensor for the selective detection of cyanide and its application in food samples. RSC Adv..

[40] Hu J.H., Sun Y., Qi J., Li Q., Wei T.B. (2017). A new unsymmetrical azine derivative based on coumarin group as dual-modal sensor for CN^−^, and fluorescent “off–on” for Zn^2+^. Spectrochim. Acta A.

[41] Wu H.L., Pan G.L., Wang H., Wang X.L., Bai Y.C., Zhang Y.H. (2014). Study on synthesis, crystal structure, antioxidant and DNA-binding of mono-, di- and poly-nuclear lanthanides complexes with bis(*N*-salicylidene)-3-oxapentane-1,5-diamine. J. Photochem. Photobiol. B Biol..

[42] Wu H.L., Bai Y.C., Zhang Y.H., Li Z., Wu M.C., Chen C.Y., Zhang J.W. (2014). Synthesis, crystal structure, antioxidation and DNA-binding properties of a dinuclear copper(II) complex with bis(*N*-salicylidene)-3-oxapentane-1,5-diamine. J. Coord. Chem..

[43] Wu H.L., Pan G.L., Bai Y.C., Wang H., Kong J. (2015). Synthesis, structure, antioxidation, and DNA-bindingstudies of a binuclear ytterbium(III) complex with bis(*N*-salicylidene)-3-oxapentane-1,5-diamine. Res. Chem. Intermed..

[44] Wu H.L., Wang C.P., Wang F., Peng H.P., Zhang H., Bai Y.C. (2015). A new manganese(III) complex from bis(5-methylsalicylaldehyde)-3-oxapentane-1,5-diamine: Synthesis, characterization, antioxidant activity and luminescence. J. Chin. Chem. Soc..

[45] Chen C.Y., Zhang J.W., Zhang Y.H., Yang Z.H., Wu H.L. (2015). Gadolinium(III) and dysprosium(III) complexes with a Schiff base bis(*N*-salicylidene)-3-oxapentane-1,5-diamine: Synthesis, characterization, antioxidation, and DNA-binding studies. J. Coord. Chem..

[46] Wu H.L., Bai Y.C., Zhang Y.H., Pan G.L., Kong J., Shi F., Wang X.L. (2014). Two lanthanide(III) complexes based on the schiff base *N*,*N*-Bis(salicylidene)-1,5-diamino-3-oxapentane: Synthesis, characterization, DNA-binding properties, and antioxidation. Z. Anorg. Allg. Chem..

[47] Wu H.L., Pan G.L., Bai Y.C., Wang H., Kong J., Shi F., Zhang Y.H., Wang X.L. (2014). Preparation, structure, DNA-binding properties, and antioxidant activities of a homodinuclear erbium(III) complex with a pentadentate Schiff base ligand. J. Chem. Res..

[48] Wang B.J., Dong W.K., Zhang Y., Akogun S.F. (2017). A novel relay-sensor for highly sensitive and selective detection of Zn^2+^/Pic^-^ and fluorescence on/off switch response of H^+^/OH^−^. Sens. Actuators B.

[49] Dong Y.J., Li X.L., Zhang Y., Dong W.K. (2017). A highly selective visual and fluorescent sensor for Pb^2+^ and Zn^2+^ and crystal structure of Cu^2+^ complex based-on a novel single-armed Salamo-type bisoxime. Supramol. Chem..

[50] Dong W.K., Ma J.C., Zhu L.C., Zhang Y., Li X.L. (2016). Four new nickel(II) complexes based on an asymmetric Salamo-type ligand: Synthesis, structure, solvent effect and electrochemical property. Inorg. Chim. Acta.

[51] Dong W.K., Lan P.F., Zhou W.M., Zhang Y. (2016). Salamo-type trinuclear and tetranuclear cobalt(II) complexes based on a new asymmetry salamo-type ligand: Syntheses, crystal structures and fluorescence properties. J. Coord. Chem..

[52] Dong W.K., Ma J.C., Zhu L.C., Zhang Y. (2016). Self-assembled zinc(II)-lanthanide(III) heteromultinuclear complexes constructed from 3-MeOsalamo ligand: Syntheses, structures and luminescent properties. Cryst. Growth Des..

[53] Dong X.Y., Kang Q.P., Jin B.X., Dong W.K. (2017). A dinuclear nickel(II) complex derived from an asymmetric Salamo-type N_2_O_2_ chelate ligand: Synthesis, structure and optical properties. Z. Naturforsch..

[54] Dong W.K., Zhang J.T., Dong Y.J., Zhang Y., Wang Z.K. (2016). Construction of mononuclear copper(II) and trinuclear cobalt(II) complexes based on asymmetric Salamo-type ligands. Z. Anorg. Allg. Chem..

[55] Dong W.K., Li X.L., Wang L., Zhang Y., Ding Y.J. (2016). A new application of Salamo-type bisoximes: As a relay-sensor for Zn^2+^/Cu^2+^ and its novel complexes for successive sensing of H^+^/OH^−^. Sens. Actuators B.

[56] Dong W.K., Zhang J., Zhang Y., Li N. (2016). Novel multinuclear transition metal(II) complexes based on an asymmetric Salamo-type ligand: Syntheses, structure characterizations and fluorescent properties. Inorg. Chim. Acta.

[57] Dong W.K., Ma J.C., Dong Y.J., Zhu L.C., Zhang Y. (2016). Di-and tetranuclear heterometallic 3d-4f cobalt(II)-lanthanide(III) complexes derived from a hexadentate bisoxime: Syntheses, structures and magnetic properties. Polyhedron.

[58] Dong W.K., Ma J.C., Zhu L.C., Zhang Y. (2016). Nine self-assembled nickel(II)-lanthanide(III) heterometallic complexes constructed from a Salamo-type bisoxime and bearing *N*- or *O*-donor auxiliary ligand: Syntheses, structures and magnetic properties. New J. Chem..

[59] Gao L., Wang F., Zhao Q., Zhang Y., Dong W.K. (2018). Mononuclear Zn(II) and trinuclear Ni(II) complexes derived from a coumarin-containing N_2_O_2_ ligand: Syntheses, crystal structures and fluorescence properties. Polyhedron.

[60] Chen L., Dong W.K., Zhang H., Zhang Y., Sun Y.X. (2017). Structural variation and luminescence properties of tri- and dinuclear Cu^II^ and Zn^II^ complexes constructed from a naphthalenediol-based bis(Salamo)-type ligand. Cryst. Growth Des..

[61] Dong W.K., Akogun S.F., Zhang Y., Sun Y.X., Dong X.Y. (2017). A reversible “turn-on” fluorescent sensor for selective detection of Zn^2+^. Sens. Actuators B.

[62] Hao J., Liu L.Z., Dong W.K., Zhang J., Zhang Y. (2017). Three multinuclear Co.(II), Zn(II) and Cd(II) complexes based on a single-armed salamo-type bisoxime: Syntheses, structural characterizations and fluorescent properties. J. Coord. Chem..

[63] Zheng S.S., Dong W.K., Zhang Y., Chen L., Ding Y.J. (2017). Four Salamo-type 3d–4f hetero-bimetallic [Zn^II^Ln^III^] complexes: Syntheses, crystal structures, and luminescent and magnetic properties. New J. Chem..

[64] Dong W.K., Zheng S.S., Zhang J.T., Zhang Y., Sun Y.X. (2017). Luminescent properties of heterotrinuclear 3d–4f complexes constructed from a naphthalenediol-based acyclic bis(salamo)-type ligand. Spectrochim. Acta Part A.

[65] Li G., Hao J., Liu L.Z., Zhou W.M., Dong W.K. (2017). Syntheses, crystal structures and thermal behaviors of two supramolecular salamo-type cobalt(II) and zinc(II) complexes. Crystals.

[66] Peng Y.D., Li X.Y., Kang Q.P., An G.X., Zhang Y., Dong W.K. (2018). Synthesis and fluorescence properties of asymmetrical salamo-type tetranuclear zinc(II) complex. Crystals.

[67] Hao J., Li L.L., Zhang J.T., Akogun S.F., Wang L., Dong W.K. (2017). Four homo- and hetero-bismetallic 3d/3d-2s complexes constructed from a naphthalenediol-based acyclic bis(salamo)-type tetraoxime ligand. Polyhedron.

[68] Wang L., Hao J., Zhai L.X., Zhang Y., Dong W.K. (2017). Synthesis, crystal structure, luminescence, electrochemical and antimicrobial properties of bis(salamo)-based Co.(II) complex. Crystals.

[69] Wang F., Gao L., Zhao Q., Zhang Y., Dong W.K., Ding Y.J. (2018). A highly selective fluorescent chemosensor for CN^−^ based on a novel bis(salamo)-type tetraoxime ligand. Spectrochim. Acta Part A.

