# Supplementary material for: A Reversible Bis(Salamo)-Based Fluorescence Sensor for Selective Detection of Cd2+ in Water-Containing Systems and Food Samples
Source: Materials (Basel). 2018 Mar 29;11(4):523. doi: 10.3390/ma11040523 (PMC5951369; doi:10.3390/ma11040523)
Supplement: Supplementary file 1 [file materials-11-00523-s001.pdf]

materials

Supplementary

A Reversible Bis(salamo)-based Fluorescence Sensor for Selective Detection of Cd^{2+} in Water-containing Systems and Food Samples

Jing Hao, Xiao-Yan Li, Yang Zhang and Wen-Kui Dong *

School of Chemical and Biological Engineering, Lanzhou Jiaotong University, Lanzhou 730070, China; haojingmm@126.com (J.H.); L1401569787@163.com (X.-Y.L.); zhangy8124@163.com (Y.Z.)

* Correspondence: dongwk@mail.lzjtu.cn

Received: 27 February 2018; Accepted: 28 March 2018; Published: 28 March 2018

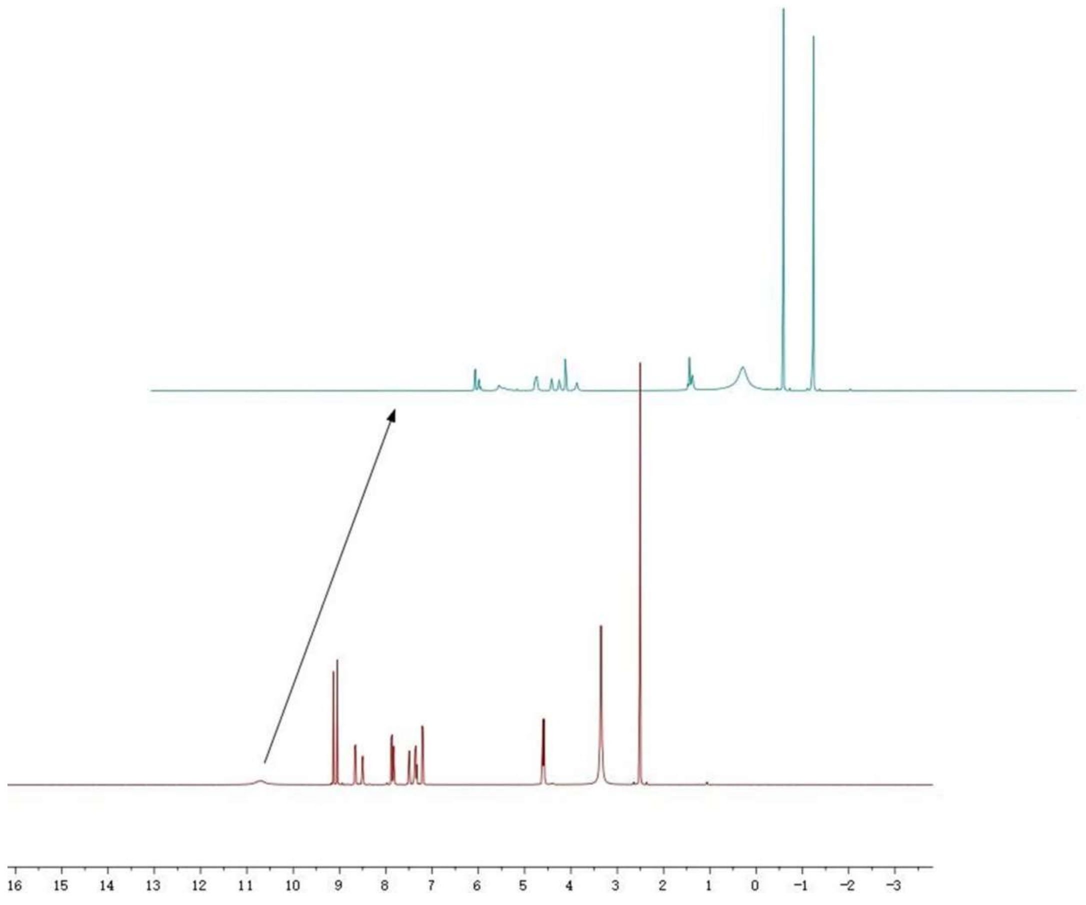

Figure S1. ^1H NMR titration in upon addition of 3.0 equiv. Cd^{2+}.

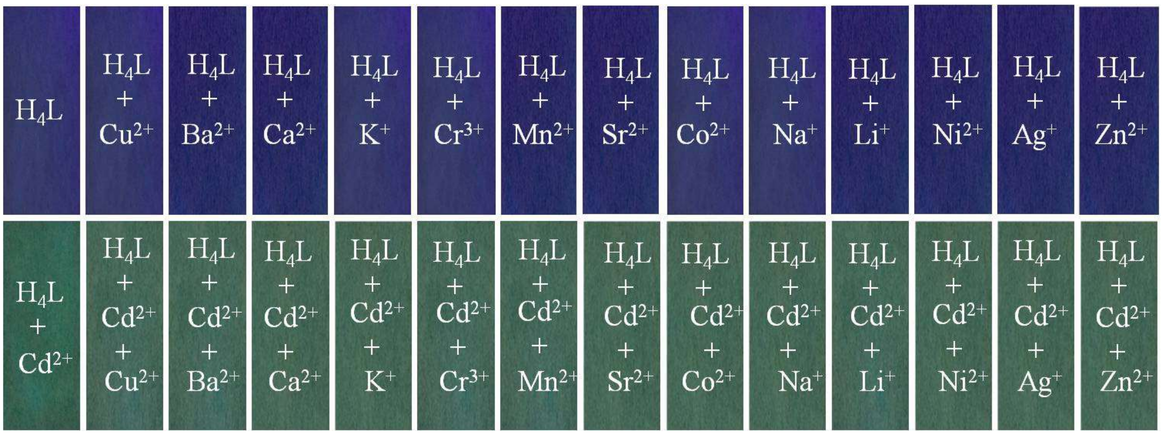

Figure S2. The result of colorimetric measured photographs with sensor H_4L for detecting Cd^{2+} under irradiation at 365 nm.
